# The use of lytic polysaccharide monooxygenases in anaerobic digestion of lignocellulosic materials

**DOI:** 10.1186/s13068-019-1611-8

**Published:** 2019-11-16

**Authors:** Thales H. F. Costa, Vincent G. H. Eijsink, Svein Jarle Horn

**Affiliations:** Faculty of Chemistry, Biotechnology and Food Science, Norwegian University of Life Sciences (NMBU), P.O. Box 5003, 1432 Aas, Norway

**Keywords:** Lytic polysaccharide monooxygenase, LPMO, Biogas, Methane, Birch, Spruce, Lignin, Biorefining

## Abstract

**Background:**

The recent discovery that LPMOs can work under anaerobic conditions when supplied with low amounts H_2_O_2_ opens the possibility of using LPMOs as enzyme aids in biogas reactors to increase methane yields from lignocellulosic materials. We have explored this possibility by studying anaerobic digestion of various lignocellulosic materials: Avicel, milled spruce and birch wood, and a lignin-rich hydrolysis residue from steam-exploded birch. The digestions were added LPMOs and various cellulolytic enzyme cocktails and were carried out with or without addition of H_2_O_2_.

**Results:**

In several cases, enzyme addition had a beneficial effect on methane production, which was partly due to components present in the enzyme preparations. It was possible to detect LPMO activity during the initial phases of the anaerobic digestions of Avicel, and in some cases LPMO activity could be correlated with improved methane production from lignocellulosic materials. However, a positive effect on methane production was only seen when LPMOs were added together with cellulases, and never upon addition of LPMOs only. Generally, the experimental outcomes showed substrate-dependent variations in process efficiency and the importance of LPMOs and added H_2_O_2_. These differences could relate to variations in the type and content of lignin, which again will affect the activity of the LPMO, the fate of the added H_2_O_2_ and the generation of potentially damaging reactive-oxygen species. The observed effects showed that the interplay between cellulases and LPMOs is important for the overall efficiency of the process.

**Conclusion:**

This study shows that it may be possible to harness the power of LPMOs in anaerobic digestion processes and improve biogas production, but also highlight the complexity of the reaction systems at hand. One complicating factor was that the enzymes themselves and other organic components in the enzyme preparations acted as substrates for biogas production, meaning that good control reactions were essential to detect effects caused by enzyme activity. As also observed during regular aerobic enzymatic digestion of lignocellulosic biomass, the type and contents of lignin in the substrates likely plays a major role in determining the impact of LPMOs and of cellulolytic enzymes in general. More work is needed to unravel the interplay between LPMOs, O_2_, H_2_O_2_, and the multitude of redox-active components found in anaerobic bioreactors degrading lignocellulosic substrates.

## Background

Biogas is an important part of current renewable fuel strategies and its usage worldwide is increasing [1]. For example, the anaerobic digestion of food waste for biomethane production has been extensively studied and is today an established process in many countries [2]. Forestry and agricultural residues have also drawn attention as potential substrates for biogas production [3]. Anaerobic digestion of such lignocellulosic residues would be an important contribution to meet future demands for renewable fuels and generally add more value to biorefining of lignocellulosic feedstocks [4–6]. Moreover, integration of biogas production into first- and second-generation bioethanol plants would increase the output of fuels. Lignin-rich residues are the main waste material in second generation ethanol biorefineries, and since only 40% of the lignin typically is used to cover factory energy demands by burning [7], leftover residues are available for different valorization strategies such as biogas production.

Lignocellulose is mainly comprised of cellulose, hemicellulose and lignin assembled in a complex matrix, which makes lignocellulosic biomasses very recalcitrant [8]. Strategies to degrade lignocellulosic materials have included chemical and mechanical pretreatments and the use of lignocellulose-active enzymes originating from wood decay microorganisms. Lytic-polysaccharide monooxygenases (LPMOs) are oxidoreductases that can cleave recalcitrant crystalline polysaccharide chains by oxidizing one of the carbons in scissile glycosidic bonds [9–11]. LPMO activity requires a reductant, such as ascorbic acid or lignin fragments [12–14] and leads to production of oxidized oligosaccharides that can be further degraded by classical hydrolytic enzymes to produce fermentable sugar monomers. Since their discovery, LPMOs have been assumed to utilize O_2_ as co-substrate [15]. However, it has recently been discovered that H_2_O_2_ is much more effective in driving the LPMO reaction [16, 17]. It is nevertheless currently debated whether under aerobic conditions LPMOs use O_2_ directly [18] or if H_2_O_2_ is produced in situ by reactions involving O_2_, reductants and possibly non-substrate-bound LPMOs [11, 16]. In any case, and most importantly, it is now clear that LPMOs can be activated under anaerobic conditions by addition of H_2_O_2_.

Anaerobic digestion (AD) is a step-wise degradation of organic matter to biogas carried out by a microbial community. Enzymatic hydrolysis of polymeric material is the first step and, therefore, potentially a rate-limiting step during biogas production. Previous studies have shown that treatment of lignocellulosic materials by commercial or in-house produced enzymes may improve biogas production from these materials in anaerobic settings [19]. However, enzyme applications in large-scale reactions are still limited due to high costs, and new approaches are needed to harness the potential of improving biogas production processes with enzymes [20]. Building on recent research in the LPMO field, in this study, we have assessed the effects of adding LPMOs or cellulolytic enzyme blends containing LPMOs on biogas production from different lignocellulosic materials. The aim was to investigate if LPMOs could be activated in biogas reactors, and if LPMO activity could improve biogas production.

## Results and discussion

### Verification of LPMO activity during anaerobic digestion conditions

To verify LPMO activity under the conditions used in anaerobic digestion (AD) reactions, AD reactions were set up with Avicel as a model substrate to screen for initial production of C4-oxidized disaccharides (Glc4gemGlc) produced by LPMOs. Three enzyme preparations were analyzed: purified *Nc*LPMO9C and the commercial cellulose cocktails Cellic^®^ Ctec2 (rich in LPMO activity; estimated to around 15% of the protein) and Celluclast 1.5 L (poor in LPMO activity) [21]. Enzymes were added at 4 mg protein per g DM. H_2_O_2_ was supplied at the start of the experiment and before sampling at 24 h. Equal volume of H_2_O was supplied to all control reactions where H_2_O_2_ was not added.

Table 1 shows that C4 oxidized products indeed were detected in some of the digestion reactions, and primarily in those containing *Nc*LPMO9C. The C4-oxidized products are unstable over time and difficult to quantify [17] and are probably continuously consumed by the microbial community in the biogas reactors. This makes a detailed quantitative interpretation of the data in Table 1 of little use. The highest levels of C4-oxidized products were detected in the samples taken after one minute (Table 1; Additional file 1: Figure S1). Also, the data in Table 1 show a clear trend in that detection of C4-oxidized products correlates with the presence of added LPMOs (*Nc*LPMO9C and Ctec2). Of note, the reactions denoted as anaerobic had only their headspace filled with nitrogen gas, explaining the formation of LPMO products in the “anaerobic” reaction not added H_2_O_2_, as some oxygen was present in the reaction liquid making in situ H_2_O_2_ formation possible [9]. Oxygen solubility in water at 37 °C is around 7 mg L^−1^ or 218 µM [22]. Most of this oxygen will be quickly consumed by the microbial community when the biogas reaction is initiated by substrate addition. However, at the start of the experiments some oxygen will be available to drive LPMO reactions.

**Table 1:** Initial Glc4gemGlc concentrations in biogas reactions with Avicel supplemented with commercial cellulase cocktails (Cellic Ctec2 or Celluclast) or purified *Nc*LPMO9C as well as H_2_O_2_

Sample	Glc4gemGlc (µM)							
	Anaerobic reactions^a^				Aerobic reactions			
	1 min	1 h	4 h	24 h	1 min	1 h	4 h	24 h
Control-Inoculum-only	nd^b^	nd	nd	nd	nd	nd	nd	nd
Control-Inoculum + H_2_O_2_	nd	nd	nd	nd	nd	nd	nd	nd
Avicel-only	nd^b^	nd	nd	nd	nd	nd	nd	nd
Avicel + H_2_O_2_	nd	nd	nd	nd	nd	nd	nd	nd
Avicel + Ctec2 + H_2_O_2_	nd	*0.6*	nd	nd	nd	*1.1*	*2.8*	nd
Avicel + Celluclast + H_2_O_2_	nd	nd	nd	nd	nd	nd	*0.8*	nd
Avicel + *Nc*LPMO9C + H_2_O_2_	*5.3*^*b*^	*1.4*	*1.4*	nd	*8.4*	*2.8*	*2.5*	nd
Avicel + Ctec2	nd	nd	nd	nd	nd	*1.4*	*2.5*	nd
Avicel + Celluclast	nd	nd	nd	nd	nd	nd	*0.8*	nd
Avicel + *Nc*LPMO9C	*9.5*^*b*^	*1.7*	*0.6*	nd	*9.8*	*3.4*	*3.1*	nd
Avicel + boiledCtec2 + H_2_O_2_	nd	nd	nd	nd	nd	nd	nd	nd
Avicel + boiledCelluclast + H_2_O_2_	nd	nd	nd	nd	nd	nd	nd	nd
Avicel + boiled*Nc*LPMO9C + H_2_O_2_	nd	nd	nd	nd	nd	nd	nd	nd

The headspace of the reactions contained nitrogen (anaerobic) or air (aerobic). The microbial inoculum was collected from a food-waste-and-cow-manure fed laboratory reactor. Enzymes were supplied once at time 0 at 4 mg of protein/g of substrate. Hydrogen peroxide was supplied at 0 h and 24 h (1 min before sampling at vigorous stirring) at 0.1 mM final concentration. The same volume of deionized water was added in all reactions without H_2_O_2_. Boiled enzyme control reactions plus H_2_O_2_ were also included. *nd* not detected

^a^Only the headspace was sparged with N_2_, meaning that some oxygen was present in the reaction mixture

^b^Chromatograms are shown in Additional file 1: Figure S1

No oxidized products were detected after 24 h for any of the reactions, which might be a result of the active microbial community metabolizing all the released oxidized products, or a gradual inactivation of the LPMOs combined with instability of earlier generated products. It is reasonable to assume that such a rich microbial community can hydrolyze Glc4gemGlc to glucose and an oxidized monomer that will be consumed by the microbes. Importantly, no C4 oxidized products were observed in reactions with added boiled enzymes, substrate-only reactions or inoculum-only reactions.

### Anaerobic digestion of lignocellulosic substrates supplemented with LPMOs

Encouraged by the initial detection of C4 oxidized products during anaerobic digestion, a larger set of experiments was carried out to find possible links between LPMO activity and methane production. These anaerobic digestion experiments were run for 40 days and included four different substrates: Avicel, milled birch wood, milled spruce wood and a lignin-rich residue (LRR) obtained upon enzymatic hydrolysis of steam-exploded birch wood (see Table 2). The same three enzyme treatments as described above were included plus a blend of 85% Celluclast and 15% *Nc*LPMO9C (Cell + 9C), all added at 4 mg protein per g DM substrate. In these experiments, H_2_O_2_ or deionized water was injected four times, at 0, 24, 48 and 72 h. The effects on methane production rates are shown in Additional file 1: Figures S2–S5. As control reactions we used boiled enzymes to account for non-enzymatic effects on biogas yield. In this way we accounted for all organic compounds added with the enzyme preparations and not only protein, which would be the case if we used a protein control such as Bovine Serum Albumin (BSA).

**Table 2:** Dry matter (DM), volatile solids (VS) and chemical composition of the lignocellulosic materials used as substrates in anaerobic reactions

Materials	DM (% w/w)	VS^b^ (% w/w)	Chemical compositon^a^ [% (w/w) of dry matter]						
			Arabinose	Galactose	Glucose	Xylose	Mannose	Total lignin	Total carbohydrates
Birch	93.5	93.2	5.0	1.1	34.5	26.3	2.6	19.3^c^	69.6
Spruce	93.4	93.0	1.3	1.9	38.0	5.6	11.5	27.0^c^	58.3
LRR birch^b^	94.5	93.9	0.0	0.1	9.1	1.1	0.1	80.3^c^	10.5

^a^Values represent sugars in their dehydrated polymeric form

^b^Lignin-rich residue recovered as insoluble fraction at the end of a 48-h enzymatic hydrolysis of steam-explosion birch with Cellic Ctec2

^c^The ratio of syringylpropane to guaiacylpropane (S/G ratio) was determined to be 2.8 and 5.8 for birch and LRR birch, respectively. For spruce only G could be quantified

**Fig. 1:**
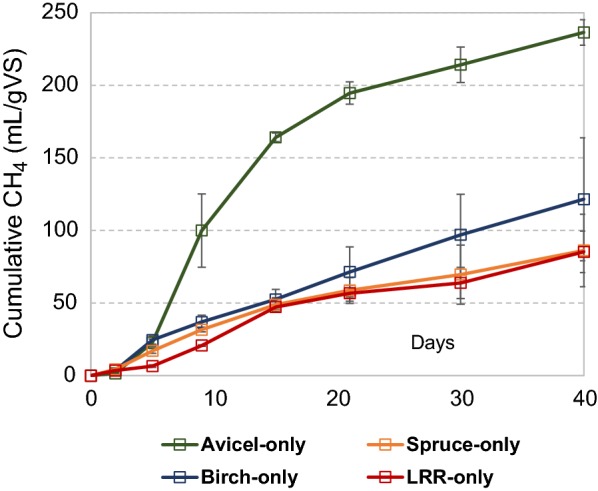
Cumulative methane production during anaerobic digestion of Avicel, birch wood, spruce wood and a lignin-rich residue from birch (LRR). The microbial inoculum included was collected from a cow-manure-and-food-waste fed biogas reactor. Background inoculum methane release was subtracted from all reactions. The curves represent the average of two separate experiments

Analysis of methane production in reactions with no added enzymes revealed that anaerobic degradation of Avicel gave 2–3 times more cumulative methane after 40 days (236 mL gVS^−1^) than the other substrates (121 mL gVS^−1^ for birch; 86 mL gVS^−1^ for spruce; 85 mL gVS^−1^ for LRR birch) (Fig. 1). Since Avicel is comprised of mainly cellulose, it is reasonable that methane production is higher for this substrate. However, it is interesting that the high levels of carbohydrates in birch (69.6% w/w) and spruce (58.3% w/w) did not translate into more methane compared to lignin-rich residue from birch (with only 10.5% w/w carbohydrates). The recalcitrance of birch and spruce might be related to the fact that these materials had not undergone any chemical or physical treatments except milling, while LRR had undergone steam explosion. Several studies have shown the benefit of chemical and/or physical treatments towards production of biogas from lignocellulosic materials [5, 23, 24]. Also, it is important to note that most of the methane produced from LRR birch at the end of 40 days was originating from the lignin fraction. Based on the theoretical methane potential for cellulose (415 mL per g VS) and assuming that the entire carbohydrate fraction from LRR birch was consumed, 82 mL per g VS of the methane was generated from lignin, which represents 96% of the total accumulated methane. A previous study with anaerobic reactions with lignin-rich residue from birch also estimated that most of cumulative methane must have been produced from the lignin-fraction after 16 days of reaction [25].

**Fig. 2:**
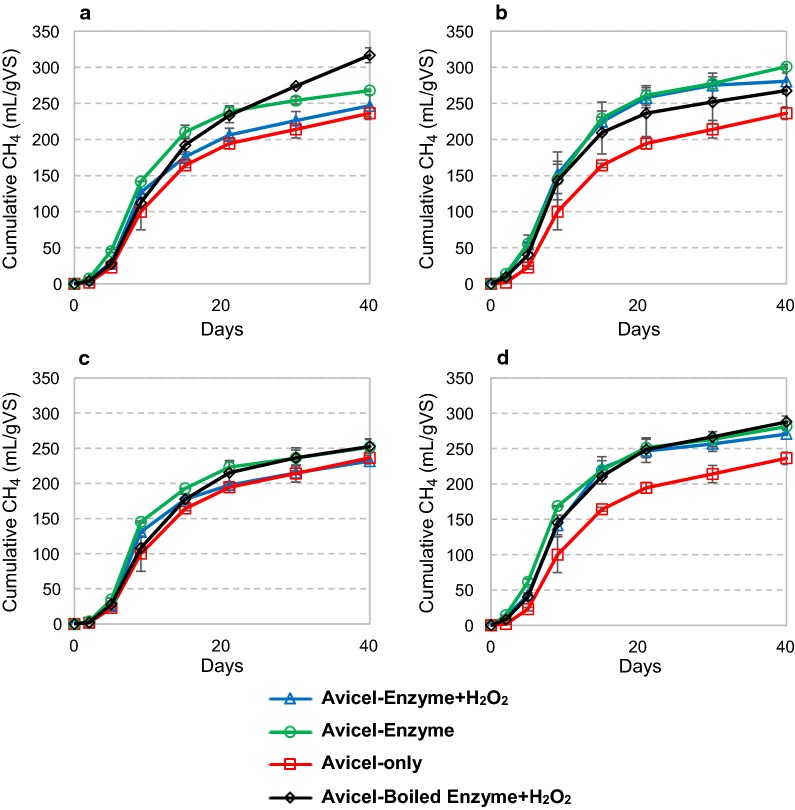
Cumulative methane production during anaerobic digestion of Avicel with addition of Cellic Ctec2 (**a**), Celluclast (**b**), *Nc*LPMO9C (**c**) and an 85% Celluclast and 15% *Nc*LPMO9C (Cell + 9C) blend (**d**). The microbial inoculum was collected from a cow-manure-and-food-waste fed biogas reactor. Enzymes were supplied once at day 0 at 4 mg of protein per gram of substrate, with and without following addition of H_2_O_2_. Where indicated, hydrogen peroxide was supplied at 0 h, 24 h, 48 h and 72 h at 0.1 mM final concentration. Deionized water was added in all reactions without H_2_O_2_. Boiled enzyme control reactions with added H_2_O_2_ are also shown. Background inoculum methane release was subtracted from all reactions in the calculation of methane production. The curves represent the average of two separate experiments. Methane production rates for these experiments are shown in Additional file 1: Figure S2

Figure 2 clearly shows that addition of the commercial enzyme preparations CTec2 (Fig. 2a) and Celluclast (Fig. 2b) leads to faster and larger biogas accumulation from Avicel, regardless of whether the enzymes were boiled or not. This shows that the commercial enzyme preparations contain compounds that are used as substrates by the microbes. This would include the enzymes themselves and other organic compounds found in the enzyme preparations. Biogas production in a control reaction with only BSA as a substrate, at the same protein concentration as for the enzyme trials, was lower than the increase in biogas production caused by addition of enzyme blends (Additional file 1: Figure S6). This clearly indicates that other compounds than protein in the enzyme preparations contribute to biogas production. It should be noted that the inoculum used for the biogas reactions had a relatively low C/N ratio meaning that the microbial communities were not limited by nitrogen.

The effect of adding enzymes on overall biogas yields was much less when using an in-house purified LPMO in buffer (Fig. 2c). Overall, Fig. 2 illustrates that these enzyme activity-independent effects dominated the observed effects of enzyme additions. Compared to boiled enzyme controls, the reactions with added Ctec2, *Nc*LPMO9C or the Cell + 9C blend did show a slightly faster methane accumulation during the first phase of the reaction (Fig. 2a, c, d; green lines; the actual rates are shown in Additional file 1: Figure S2). This effect was not observed when H_2_O_2_ was supplied. Generally, addition of H_2_O_2_ had no effect or a negative effect on final biogas accumulation (compare green and blue curves in Fig. 2). Boiling of the enzymes had little effect on final methane yields from Avicel, except for CTec2 reactions where boiled enzyme gave a higher final methane yield (Fig. 2a). There is no obvious explanation for this observation; it is possible that the boiling made proteins and other compounds in the Ctec2 preparation more accessible to subsequent microbial digestion.

**Fig. 3:**
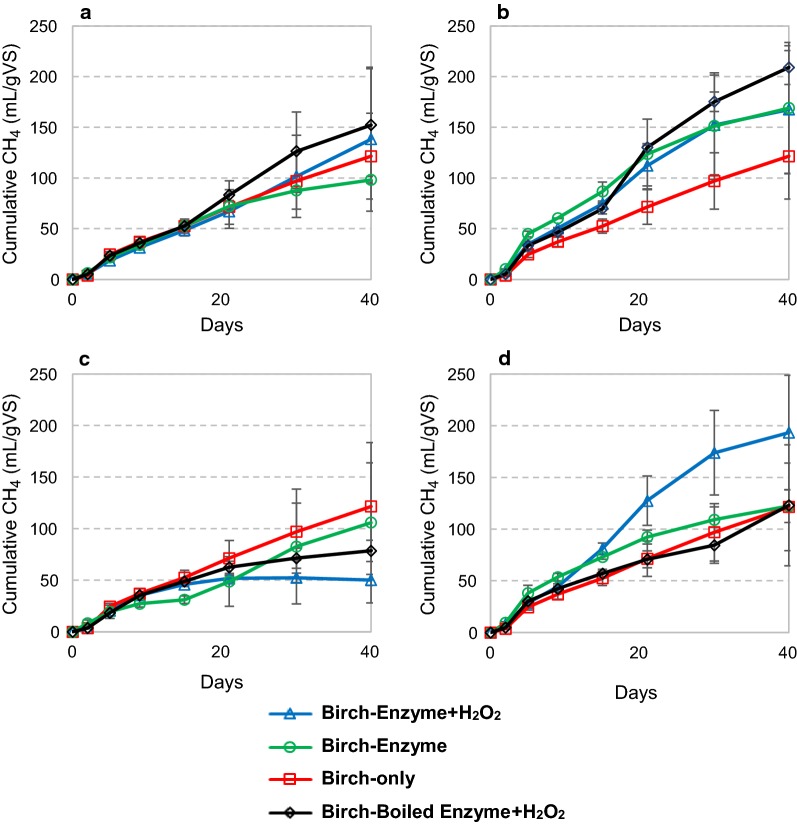
Cumulative methane production during anaerobic digestion of birch with addition of Cellic Ctec2 (**a**), Celluclast (**b**), *Nc*LPMO9C (**c**) and an 85% Celluclast and 15% *Nc*LPMO9C (Cell + 9C) blend (**d**). The microbial inoculum was collected from a cow-manure-and-food-waste fed biogas reactor. Enzymes were supplied once at day 0 at 4 mg of protein per gram of substrate, with and without following addition of H_2_O_2_. Where indicated, hydrogen peroxide was supplied at 0 h, 24 h, 48 h and 72 h at 0.1 mM final concentration. Deionized water was added in all reactions without H_2_O_2_. Boiled enzyme control reactions with added H_2_O_2_ are also shown. Inoculum methane release was subtracted from all samples in the calculation of methane production. The curves represent the average of two separate experiments. Methane production rates for these experiments are shown in Additional file 1: Figure S3

Addition of enzymes to biogas reactions with milled birch yielded varying results (Fig. 3) not allowing general conclusions to be drawn concerning the effect of adding enzymes and/or H_2_O_2_. In this case, only Celluclast showed some tendencies of being beneficial for the biogas process. Interestingly, addition of Cell + 9C and H_2_O_2_ resulted in a clear improvement in methane accumulation, relative to the same reactions with boiled enzyme or without H_2_O_2_ (Fig. 3d). After 40 days of reaction, the biogas production was 57% higher than in the reactions with boiled enzyme. After 40 days, the biomethane potential for the birch samples plus enzymes, with and without H_2_O_2_, respectively, was 138 and 98 mL gVS^−1^ for Ctec2, 169 and 168 mL gVS^−1^ for Celluclast, 50 and 106 mL gVS^−1^ for *Nc*LPMO9C, and 193 and 122 mL gVS^−1^ for Cell + 9C.

**Fig. 4:**
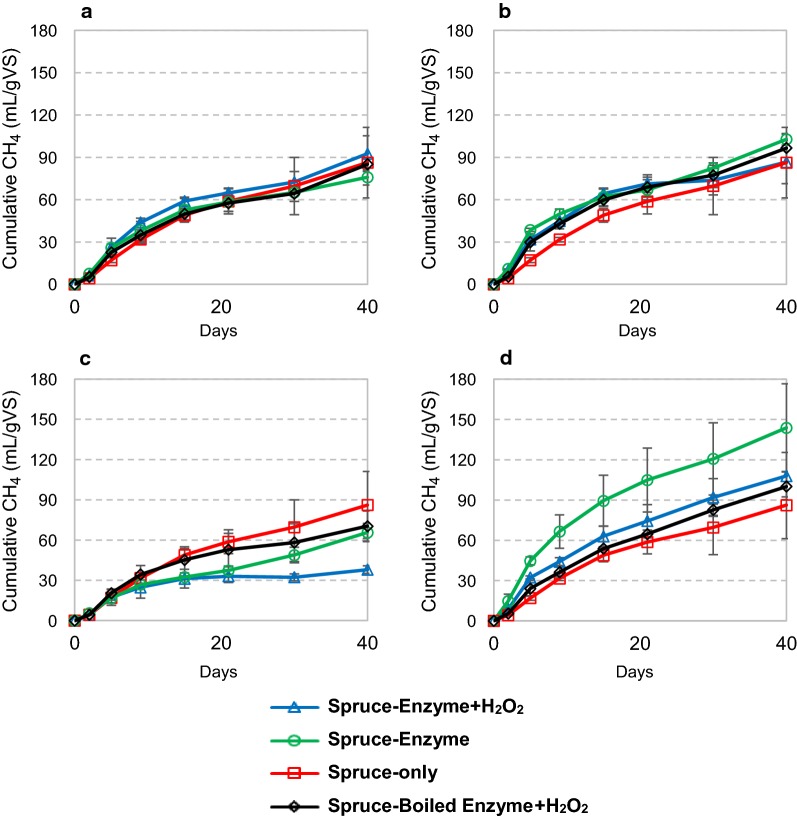
Cumulative methane production during anaerobic digestion of spruce with addition of Cellic Ctec2 (**a**), Celluclast (**b**), *Nc*LPMO9C (**c**) and an 85% Celluclast and 15% *Nc*LPMO9C (Cell + 9C) blend (**d**). The microbial inoculum was collected from a cow-manure-and-food-waste fed biogas reactor. Enzymes were supplied once at day 0 at 4 mg of protein per gram of substrate, with and without following addition of H_2_O_2_. Where indicated, hydrogen peroxide was supplied at 0 h, 24 h, 48 h and 72 h at 0.1 mM final concentration. Deionized water was added in all reactions without H_2_O_2_. Boiled enzyme control reactions with added H_2_O_2_ are also shown. Inoculum methane release was subtracted from all samples in the calculation of methane production. The curves represent the average of two separate experiments. Methane production rates for these experiments are shown in Additional file 1: Figure S4

Anaerobic reactions with spruce as substrate (Fig. 4) showed tendencies of a somewhat faster methane accumulation within the first 15 days of incubation, especially for Celluclast (compared to reactions with boiled enzyme). However, enzyme addition did not affect final methane yields, which with and without H_2_O_2_, respectively, were 93 and 76 mL gVS^−1^ for Ctec2, and 87 and 103 mL gVS^−1^ for Celluclast after 40 days. *Nc*LPMO9C additions did not benefit methane production and led to inhibition when combined with H_2_O_2_ (Fig. 4c). However, combination of *Nc*LPMO9C and Celluclast led to a clear enhancement in biomethane production, in particular for the reactions without H_2_O_2_ (Fig. 4d), which reached a final methane yield of 144 mL gVS^−1^. This yield after 40 days is 44% higher compared to the control reactions with boiled enzyme.

**Fig. 5:**
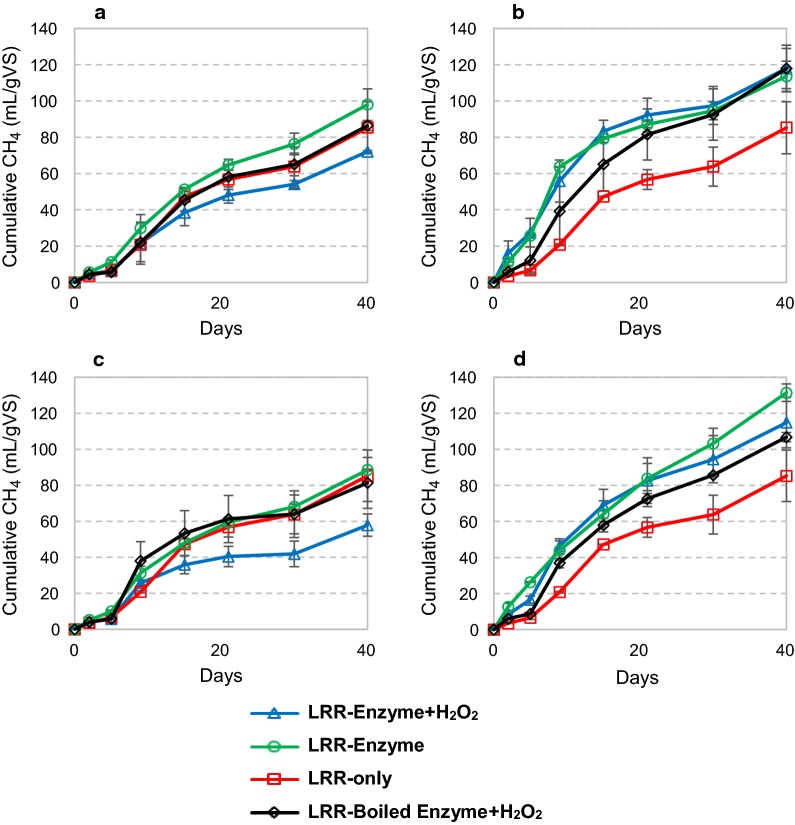
Cumulative methane production during anaerobic digestion of lignin-rich residue from birch (LRR) with addition of Cellic Ctec2 (**a**), Celluclast (**b**), *Nc*LPMO9C (**c**) and an 85% Celluclast and 15% *Nc*LPMO9C (Cell + 9C) blend (**d**). The microbial inoculum was collected from a cow-manure-and-food-waste fed reactor. Enzymes were supplied once at day 0 at 4 mg of protein per gram of substrate, with and without following addition of H_2_O_2_. Where indicated, hydrogen peroxide was supplied at 0 h, 24 h, 48 h and 72 h at 0.1 mM final concentration. Deionized water was added in all reactions without H_2_O_2_. Boiled enzyme control reactions with added H_2_O_2_ are also shown. Inoculum methane release was subtracted from all samples in the calculation of methane production. The curves represent the average of two separate experiments. Methane production rates for these experiments are shown in Additional file 1: Figure S5

Reactions containing lignin-rich residue from birch (LRR) also showed a tendency of faster initial methane accumulation upon addition of cellulase cocktails, again particularly for Celluclast, but this time more independent of the addition of H_2_O_2_ (Fig. 5). After 40 days of incubation, the Ctec2 reactions had produced 72 and 98 mL gVS^−1^ of cumulative methane (Fig. 5a), with and without H_2_O_2_, respectively, while Celluclast addition gave 118 and 114 mL gVS^−1^ (Fig. 5b). Interestingly, as observed for spruce (Fig. 4), the Cell + 9C combination resulted in both higher initial methane production rates (Additional file 1: Figure S5D) and final methane yields (Fig. 5d). The reaction with H_2_O_2_ addition reached 115 mL gVS^−1^ of cumulative methane after 40 days. The improvement in methane release was more evident when H_2_O_2_ was not supplied, with a maximum enhancement, relative to boiled control reaction, of 200% at day 5 (26,4 mL gVS^−1^), and 23% after 40 days (131 mL gVS^−1^; Fig. 5d). It is interesting to note that this increased production of methane happened even though the total polysaccharide content is low in LRR (10.5%; Table 1). LPMOs are known for acting on the crystalline portions of polysaccharide substrates [26]. This feature would be highly relevant for degrading the residual polysaccharides in lignin-rich residues like LRR, as these would likely contain recalcitrant crystalline structures remaining after enzymatic hydrolysis.

As observed for the reactions with other lignocellulosic substrates, addition of *Nc*LPMO9C alone had little effect on methane production from LRR (Fig. 5c). The combination of adding *Nc*LPMO9C and H_2_O_2_ led to reduced methane production (Fig. 5c), similar to that which was observed in the reactions with spruce (Fig. 4c) and birch (Fig. 3c), but different from that observed in the Avicel reactions (Fig. 2c). An important difference between Avicel and the other substrates is that Avicel does not contain lignin. Also, since *Nc*LPMO9C was a purified enzyme preparation, reactions with *Nc*LPMO9C contained the highest concentration of LPMOs (4 mg/g). It is well known that LPMOs can be reduced and activated by the redox power of aromatic compounds, such as lignin [14, 27, 28], which activates the LPMO and changes the nature of the (now oxidized) lignin [29]. Activated LPMOs, i.e. LPMOs in the copper Cu(I) state, may react non-productively with the added H_2_O_2_, which will generate reactive oxygen species that may damage the LPMOs themselves or other enzymes and microbes in the reaction [30]. Lignin modification (i.e. oxidation) by LPMOs may potentially also affect the reactivity of lignin with H_2_O_2_, potentially generating damaging compounds, such as reactive oxygen species.

Overall, the experiments described above do show improvements of methane production rates and methane yields in some settings (relative to boiled enzyme), but without providing a very clear picture of what is optimal. The effects on methane production rates are shown in Additional file 1: Figures S2–S5 and may seem more pronounced than the effects on final methane yields. The largest effects were typically observed in the beginning of the reactions and mostly when H_2_O_2_ was not supplied. The fact that most of the enzymatic enhancement occurred in the first days of the reactions coincides with the notion that the enzymatic hydrolysis of polysaccharides may be rate-limiting in the early stages of an anaerobic digestion [23, 31, 32], especially if we consider that the concentration of any solubilized oxygen would be higher in the beginning of reactions, allowing LPMO activation.

In general, Celluclast additions showed the largest effects on methane yields after 40 days. The addition of Cellic Ctec2, which is a modern cellulase cocktail containing LPMOs that should be capable of more efficiently degrading cellulose, at least in the presence of H_2_O_2_ [17, 21], tended to work less well, compared to Celluclast. An explanation could be that Celluclast contains more compounds that can be used for methane production. On a volume basis we also added more Celluclast to the biogas reactions since the enzyme concentration in this enzyme blend was lower than in Cellic Ctec2 (see “Enzyme preparations” section). Interestingly, Cell + 9C led to the largest enhancements in biomethane production. Previous studies have demonstrated that supplementing Celluclast with LPMOs improves saccharification of lignocellulosic materials from spruce and birch and that this improvement was linked to LPMO activity [21, 33]. It is plausible that this is the case also in biogas reactors, but more experiments are needed to confirm this. Importantly, increased methane yields were shown only for lignocellulosic substrates and not for Avicel, indicating that the presence of lignin is important. The lignin can affect the reactions by not only serving as a reductant needed for LPMO activation, but also affecting H_2_O_2_ concentrations in the suspension by either reacting directly with H_2_O_2_ or, if O_2_ is present [22], result in in situ H_2_O_2_ production [9]. The type and origin of the lignin, as well as lignin modifications due to steam-explosion treatment and enzymatic hydrolysis, could play a role in how the various lignocellulosic materials interact with LPMOs [34] and H_2_O_2_, which could explain the variations observed in the present study. The spruce lignin consisted mainly of guaiacyl units, while the birch and LRR-birch had syringyl-guaiacyl (S/G) ratios of 2.8 and 5.8, respectively (see Table 2). It is known that lignin can generate electrons and interact with LPMOs [29], and it has been shown that methoxylated and methylated diphenols get oxidized due to LPMO activity [28]. Thus, the substrates used in this study have clear difference in the lignin composition, but currently it is not known in detail how this influences LPMO activity.

The positive effects of the Cell + 9C mixture on methane production in some settings show that the cellulolytic system is complex and demands several enzymes to function properly. It is intriguing that the Cell + 9C blend has a better effect on biogas production than Ctec2, which also is a cellulase/LPMO mixture. This could indicate that not only the cellulase/LPMO ratio is important, but also the specific enzymes in the cocktails. It is not known which LPMOs are included in Ctec2, but it has been shown that the cocktail, similarly to *Nc*LPMO9C, also produces C4 oxidized sugars (Table 1) [21]. Nonetheless, LPMOs might differ in their affinity to the substrate, as well as in their reactivity with H_2_O_2._

The observed effects of adding H_2_O_2_ varied. In most cases, supply of H_2_O_2_ was not beneficial. As H_2_O_2_ was supplied only once a day, peak concentrations were as high as 0.100 mM, which may be toxic to the microorganisms and may promote the potentially damaging LPMO-catalyzed side reactions discussed above. The difference between birch (Fig. 3) and spruce (Fig. 4) in reactions with the Cell + 9C mixture is remarkable. For birch, addition of H_2_O_2_ led to increased methane yields, whereas for spruce the reaction without addition of H_2_O_2_ had the highest methane yield. This might be related to the large difference in the content of S and G in the lignin fraction of these substrates (see Table 2), and thus possible different reactivity towards H_2_O_2_. It should be noted that this difference between spruce and birch was confirmed by additional analytics, as discussed below.

### LPMO activity in digestions of lignocellulosic substrates

In order to confirm the activity of *Nc*LPMO9C in reactions with lignocellulosic substrates, the formation of C4 oxidized products (Glc4gemGlc) during the first 24 h of anaerobic digestions was analyzed. Milled birch, milled spruce and LRR birch were used as substrates. Only the two enzyme conditions involving *Nc*LPMO9C were included in this analysis: addition of purified *Nc*LPMO9C or addition of the Cell + 9C blend. H_2_O_2_ was injected at 0 h and 24 h at a final concentration of 0.1 mM. Table 3 shows that oxidized products were detected for some of the conditions. Reactions with added *Nc*LPMO9C showed oxidized products after 1 min and 1 h, but only for birch. After 4 h, similar amounts of oxidized products were detected in four reactions: with spruce and no H_2_O_2_ supplied, and with birch with H_2_O_2_ supplied, in reactions added *Nc*LPMO9C or Cell + 9C (Table 3; see Additional file 1: Figure S7 for chromatograms). After 24 h, small amounts of C4 oxidized products were detected in all samples, but one.

**Table 3:** Initial Glc4gemGlc concentrations in biogas reactions with birch, spruce and lignin-rich residue from birch (LRR) supplemented with purified *Nc*LPMO9C or 85% Celluclast plus 15% *Nc*LPMO9C (Cell + 9C)

Sample:^a^	Glc4gemGlc (µM)			
	1 min	1 h	4 h	24 h
Birch-*Nc*LPMO9C + H_2_O_2_	*3.9*	*0.3*	*2.5*^b^	*0.6*
Birch + *Nc*LPMO9C	*3.4*	*0.3*	nd	*0.6*
Spruce + *Nc*LPMO9C + H_2_O_2_	nd	nd	nd	*0.8*
Spruce + *Nc*LPMO9C	nd	nd	*2.5*^b^	nd
LRR + *Nc*LPMO9C + H_2_O_2_	nd	nd	nd	*0.3*
LRR + *Nc*LPMO9C	nd	nd	nd	*0.6*
Birch- Cell + 9C +H_2_O_2_	nd	nd	*2.8*^b^	*0.8*
Birch + Cell + 9C	nd	nd	nd	*1.1*
Spruce + Cell + 9C +H_2_O_2_	nd	nd	nd	*1.1*
Spruce + Cell + 9C	nd	nd	*2.0*^b^	*0.8*
LRR + Cell + 9C + H_2_O_2_	nd	nd	nd	*0.8*
LRR + Cell + 9C	nd	nd	nd	*0.8*

The microbial inoculum was collected from a food-waste-and-cow-manure fed reactor. Enzymes were supplied once at time 0 at 4 mg of protein/g of substrate, with and without following addition of H_2_O_2_. Hydrogen peroxide was supplied at 0 h and 24 h (1 min before sampling at vigorous stirring) at 0.1 mM final concentration. Deionized water was added in all reactions without H_2_O_2_. *nd* not detected

^a^Only headspace was sparged with N2

^b^Chromatograms are shown in Additional file 1: Figure S6

Importantly, the remarkable contrast between the effect of H_2_O_2_ on reactions with birch or spruce is reflected in methane production in the reactions with Cell + 9C (Figs. 3d and 4d). In parallel with the detected LPMO activities (Table 3), the birch biogas reaction was most efficient with supplied H_2_O_2_ (Fig. 3d), whereas the spruce reaction was most efficient in the absence of added H_2_O_2_. When supplemented with just *Nc*LPMO9C or Celluclast, no increase in methane production was observed (Figs. 3 and 4b–c). This shows that even though *Nc*LPMO9C was active in the anaerobic reactions with spruce and birch, addition of extra cellulases was necessary to translate the LPMO activity into increased methane production. Detection of C4 oxidized products after 24 h was higher in all conditions with Cell + 9C, compared to *Nc*LPMO9C only (Table 3). This indicates synergy between Celluclast and *Nc*LPMO9C, maybe due to a more balanced LPMO/cellulase ratio.

## Conclusions

This study provides data on methane production from different lignocellulosic substrates. Interestingly, methane could also be produced from a lignin-rich hydrolysis residue, indicating that the lignin fraction contained biologically available carbon. The beneficial effect of adding boiled enzyme preparations on methane formation indicates that compounds present in the enzyme preparations can be used for methane production. This effect was more visible for the “clean” cellulosic substrate Avicel, compared to natural, more complex, lignocellulosic substrates.

This study also shows for the first time that purified LPMOs or LPMOs in cellulolytic blends can be activated in anaerobic biogas reactors. LPMO activation took place irrespective of H_2_O_2_ supply, indicating that traces of O_2_ present at the start of anaerobic reactions were enough to drive the reactions. LPMO activity did correlate with increased methane production in some, but not all settings. H_2_O_2_ supply did not seem necessary in most of the conditions and was in fact unfavorable in some reactions. The presence of lignin in the substrates seemed to contribute to reduced methane yields in reactions with H_2_O_2_ and excess of LPMOs. Possible explanations for these observations are discussed above.

The impact of LPMOs on (aerobic) enzymatic conversion of cellulose by cellulase cocktails is undisputed [21, 26, 33, 35]. The present data show that it may be possible to also harness LPMO power in anaerobic digestion processes but also highlight the enormous complexity of both the enzymes themselves and the reaction systems that we are working with (see also [36] for a recent discussion). Much more work is still needed to unravel the interplay between LPMOs, O_2_, H_2_O_2_, and the multitude of redox-active components in a lignocellulose containing biogas reactor. Instead of manual addition of H_2_O_2_, the use of gradual pumping of H_2_O_2_ should be explored.

## Methods

### Raw materials, substrate preparations and chemicals

Avicel^®^ PH-101 (~ 50 μm particles; St. Louis, USA) was purchased from Sigma-Aldrich and used as is. Spruce (*Picea abies*) and birch (*Betula pubescens*) materials were collected from local trees harvested in Norway in 2016. Trees were debarked, processed into wood chips and oven dried at 50 C. The wood chips were milled using a knife mill (SM 2000, Retsch, Haan, Germany) to 6 mm particle size and then stored at room temperature and dry conditions. Milled spruce and birch used in the experiments were prepared by further milling the 6-mm particle size materials in the knife mill and sieving the biomass through a 1-mm sieve. The 1 mm-particle-size birch and spruce were stored at room temperature and dry conditions until further use. Steam-exploded birch (SE birch) was prepared using a steam-explosion unit produced by Cambi AS (Asker, Norway) and 6 mm particle-size birch. Optimal steam-explosion conditions for birch, established previously for biogas reactions [23], were used (210 °C and 10 min residence time).

Lignin-rich residues (LRR) from birch were prepared by hydrolyzing the SE birch with Cellic^®^ Ctec2 (4 mg protein g DM^−1^) for 48 h. The enzymatic hydrolysis was conducted in sodium acetate buffer (50 mM, pH 5.0) in a borosilicate 5L bottle with 3L working volume and 10% SE birch (w/w DM). At the end of hydrolysis, the hydrolysate, including insoluble residues, was transferred and split into large centrifuge containers and spun down at 15,900*g* for 15 min (Beckman Coulter, Avanti J-26S XP; Brea, CA, USA). Supernatants containing hydrolysate were disposed and the insoluble pellet was washed at least five times by adding deionized water and repeating the centrifugation process and supernatant disposal. Washed insoluble residues were pooled together and kept under 30 °C with ventilation until no visible liquid was present (approximately 48 h). The residues were then moved to room-temperature to finish air-drying for at least 5 days. The dried LRR was pulverized by a mortar and pestle and sieved to pass a 1-mm sieve. The lignin-rich materials were stored at room-temperature and dry conditions.

Dry matter and volatile solids of all materials were determined according to standard method [37]. Avicel was considered to have a 100% (w/w) VS. DM and VS for the lignocellulosic materials are shown in Table 2.

Other chemicals included in this study were purchased from Sigma-Aldrich unless specified otherwise.

### Composition analysis

The chemical composition of the lignocellulosic materials was determined according to the sulfuric-acid hydrolysis standard method by NREL [38]. (Table 2). Monosaccharides from composition analysis were detected by High-Performance Anion-exchange Chromatography (HPAEC) using a Dionex ICS 3000 system (Dionex, Sunnivale, CA, USA) equipped with a CarboPac PA1 column operated at 30 °C and with a pulsed amperometric detector (PAD). Sugars were eluted isocratically with 1 mM KOH at 0.25 mL min^−1^ flow rate. For analysis of syringylpropane and guaiacylpropane ^1^H-^13^C heteronuclear single quantum coherence (HSQC) nuclear magnetic resonance (NMR) analysis was applied, as described in detail previously [25].

### Enzyme preparations

Cellic^®^ CTec2 and Celluclast 1.5L were provided by Novozymes A/S (Bagsværd, Denmark). An LPMO from *Neurospora crassa* (*Nc*LPMO9C) was expressed and purified as described by Müller et al. [21]. Protein concentrations in all enzyme preparations was determined using the Bio-Rad Protein Assay (Bio-Rad, USA), which is based on the Bradford method [39], using Bovine Serum Albumin (BSA) as standard. Cellic Ctec2 contained 109 mg/mL protein while Celluclast contained 26.7 mg/mL. A mixture with 85% Celluclast 1.5L and 15% purified *Nc*LPMO9C (Cell + 9C) was prepared based on protein ratio and used where needed. Boiled enzymes were prepared by incubating the enzymes at 100 °C for at least 15 min.

### Microbial inoculums

A mesophilic microbial inoculum was utilized in all experiments. The inoculum was collected from a full-scale continuously stirred tank reactor (CSTR) running with food waste and cow manure at 37 °C. Inoculums were sieved to pass a 1-mm sieve and pre-incubated for at least 7 days at 37 °C to lower the level of endogenous biogas release. The volatile solid content of the inoculum was 1.5% (w/w, based on wet weight) and the pH was approximately 7.8. Previous analyses of this inoculum have shown that the total nitrogen content is 1.0 g/L and that the C/N ratio is 11.6 [40].

### Small-scale biogas reactions

Biogas reactions were carried out in 50 mL serum bottles at 30 mL working volume. The inoculum volume was 5 mL for all samples and the inoculum-substrate ratio was 1:1 (VS:VS). Substrates and deionized water were added to each bottle separately to reach a total volume of 30 mL and achieve a final substrate concentration of 2.3 g VS/L. The starting pH was corrected to 7.0 with 0.1 M HCl in all reactions. The serum bottles were sealed with a rubber septum and a crimped aluminum ring. Headspaces were then flushed with nitrogen gas for at least 3 min with venting, replacing the air with nitrogen. Any overpressure was released afterwards before incubation. Bottles were incubated at 37 °C at 100 RPM in a shaker (Multitron Standard, Infors HT, Switzerland) for 40 days. Control reactions included inoculum-only biogas reactions.

Enzymes (native and boiled) were injected once at 4 mg protein per gram of substrate (DM) after the bottles had been incubated at 37 °C for 5 min, resulting in a final protein concentration of 9.2 mg/L or 1.5 mg nitrogen/L. This was a negligible addition of nitrogen compared to the nitrogen supplied with the inoculum (1.0 g/L × 5/30 = 0.17 g/L). Bottles were vigorously stirred by hand after enzyme addition for 3 s. H_2_O_2_ was added 1 min after enzyme addition at 0.1 mM final concentration, and again at 24 h, 48 h and 72 h. Deionized water was always added at the same volume as H_2_O_2_ to conditions without H_2_O_2_ supply. All biogas experiments were conducted in duplicate.

### Biogas analysis and calculation

The biogas composition of all reactions was monitored over 40 days. Overpressure in the headspace was measured periodically by a digital pressure transducer (GMH 3161, Greisinger Electronic, Germany) and biogas analysis was conducted when overpressure was at least around 70 kPa in the bottles. The biogas composition was assessed using a gas chromatograph (3000 Micro GC, Agilent Technologies, USA) equipped with a thermal conductivity detector (TCD), allowing detection of methane, carbon-dioxide and nitrogen gas. Overpressure in the headspace was released after GC analysis when necessary. Methane and carbon dioxide concentrations were calculated by combining the GC values with the measured overpressure and the headspace volume of the bottles, using the ideal gas law. Methane accumulation is reported at standard temperature and pressure (0 °C and 1 atm) after subtracting endogenous biogas production from the inoculum-only control reactions.

### Analysis of oxidized products

Additional reactions set up exactly as described in “Small-scale biogas reactions” section (i.e., with the same inoculum), were run for the purpose of analyzing the formation of the LPMO product 4-keto-cellobiose (Glc4gemGlc). These reactions were run for 24 h. The first test included Avicel as the sole substrate and Cellic^®^ Ctec2, Celluclast or purified *Nc*LPMO9C as enzymes. The experimental layout is shown in Table 1. Samples with an aerobic headspace (no nitrogen flushing) were also included in this test. The second test included birch, spruce and LRR as substrates and purified *Nc*LPMO9C and the Cell + 9C blend as enzymes. The experimental layout is shown in Table 3. Control reactions with boiled enzymes, inoculum alone or inoculum plus substrate only were included for the first test. H_2_O_2_ or deionized water were added twice to reactions, at 0 h and 24 h (1 min before sampling at vigorous stirring).

Sampling was carried out by collecting 300 µL of the suspension with a syringe through the septum after 1 min, 1 h, 4 h and 24 h of reaction. Aliquots were transferred to 1.5 mL tubes and kept on ice at all times, unless indicated otherwise. Aliquots were centrifuged for 3 min at 21,000*g* (Thermo Scientific) and 4 °C to separate solid and liquid fractions. Approximately 150 µL of supernatant was transferred to 96-well filter plates with 0.45 µm porosity (Thermo Scientific) and vacuum-filtered. The filtered aliquots were transferred to chromatography vials. Glc4gemGlc was detected by HPAEC using a Dionex ICS 5000 system (Dionex, Sunnivale, CA, USA) equipped with a CarboPac PA1 column at 30 °C and pulsed amperometric detection (PAD). Oxidized oligosaccharides were separated as a gradient was applied with increasing concentration of sodium acetate, as previously described by Westereng et al. [41]. Glc4gemGlc standards were prepared by using *Nc*LPMO9C and cellopentaose as described by Müller et al. [21].

## Supplementary information

**Additional file 1: Figure S1**. (A) Chromatogram of soluble fractions of anaerobic digestion reactions with Avicel supplemented with purified *Nc*LPMO9C as well as H_2_O_2_. Samples were taken initially in reactions supplemented with purified *Nc*LPMO9C, after addition of H_2_O_2_ or H_2_O. Shoulder peaks representing Glc4gemGlc, seen in reactions with *Nc*LPMO9C only, are highlighted by the dashed lines. (B) Chromatogram of a standard sample (0.001 g / L) obtained after treating cellopentaose with *Nc*LPMO9C, run together with other samples. **Figure S2**. Rate of methane accumulation during anaerobic digestion of Avicel with addition of enzymes Cellic Ctec2 (A), Celluclast (B), *Nc*LPMO9C (C) and a blend of 85 % Celluclast and 15 % *Nc*LPMO9C (D). Enzymes were supplied once at day 0 at 4 mg of protein per gram of substrate, with or without following addition of H_2_O_2_, as indicated in the figure. Hydrogen peroxide was supplied at 0h, 24h, 48h and 72h at 0.1 mM final concentration. Deionized water was added in all reactions without H_2_O_2_. Control reactions with boiled enzyme are also shown. Methane release by the inoculum methane was subtracted from all samples prior to calculation of methane production. The curves represent the average of two separate experiments. **Figure S3**. Rate of methane accumulation during anaerobic digestion of birch with addition of enzymes Cellic Ctec2 (A), Celluclast (B), *Nc*LPMO9C (C) and a blend of 85 % Celluclast and 15 % *Nc*LPMO9C (D). Enzymes were supplied once at day 0 at 4 mg of protein per gram of substrate, with or without following addition of H_2_O_2_, as indicated in the figure. Hydrogen peroxide was supplied at 0h, 24h, 48h and 72h at 0.1 mM final concentration. Deionized water was added in all reactions without H_2_O_2_. Control reactions with boiled enzyme are also shown. Methane release by the inoculum methane was subtracted from all samples prior to calculation of methane production. The curves represent the average of two separate experiments. **Figure S4**. Rate of methane accumulation during anaerobic digestion of spruce with addition of enzymes Cellic Ctec2 (A), Celluclast (B), *Nc*LPMO9C (C) and a blend of 85 % Celluclast and 15 % *Nc*LPMO9C (D). Enzymes were supplied once at day 0 at 4 mg of protein per gram of substrate, with or without following addition of H_2_O_2_, as indicated in the figure. Hydrogen peroxide was supplied at 0h, 24h, 48h and 72h at 0.1 mM final concentration. Deionized water was added in all reactions without H_2_O_2_. Control reactions with boiled enzyme are also shown. Methane release by the inoculum methane was subtracted from all samples prior to calculation of methane production. The curves represent the average of two separate experiments. **Figure S5**. Rate of methane accumulation during anaerobic digestion of lignin-rich residue from birch (LRR) with addition of enzymes Cellic Ctec2 (A), Celluclast (B), *Nc*LPMO9C (C) and a blend of 85 % Celluclast and 15 % *Nc*LPMO9C (D). Enzymes were supplied once at day 0 at 4 mg of protein per gram of substrate, with or without following addition of H_2_O_2_, as indicated in the figure. Hydrogen peroxide was supplied at 0h, 24h, 48h and 72h at 0.1 mM final concentration. Deionized water was added in all reactions without H_2_O_2_. Control reactions with boiled enzyme are also shown. Methane release by the inoculum methane was subtracted from all samples prior to calculation of methane production. The curves represent the average of two separate experiments. **Figure S6.** Control reactions showing cumulative methane production during anaerobic digestion of Bovine Serum Albumin (BSA) with and without addition of H_2_O_2_ (methane production from inoculum-only has been subtracted). As for all the other reactions with enzyme addition, the load of protein at day 0 was 9.2 mg/L of protein. H_2_O_2_ was supplied at 0h, 24h, 48h and 72h at 0.1 mM final concentration. Deionized water was added in all reactions without H_2_O_2_. The methane production was divided by the same amount of substrate (0.07 g VS) used in all reactions with lignocellulosic substrates for direct comparison. The curves represent the average of two separate experiments. **Figure S7**. (A) Chromatogram of soluble fractions of anaerobic digestion reactions with birch and spruce, supplemented with purified *Nc*LPMO9C or the blend of 85 % Celluclast and 15 % *Nc*LPMO9C (Cell+9C) as well as H_2_O_2_ or H_2_O, under anaerobic conditions. The samples shown were taken after 4h of incubation from reactions. Peaks representing Glc4gemGlc for this run are highlighted by the dashed lines. (B) Chromatogram of a standard sample (0.001 g / L) obtained after treating cellopentaose with *Nc*LPMO9C.

## Data Availability

Additional data are available from the corresponding author on reasonable request.

## Abbreviations

Cell + 9C: 85% Celluclast plus 15% *Nc*LPMO9C blend (protein ratio)
DM: dry matter
Glc4gemGlc: 4-hydroxy-*β*-d-xylo-hexopyranosyl-(1 → 4)-*β*-d-glucopyranosyl
HPAEC: high-performance anion exchange chromatography
HPLC: high-performance liquid chromatography
LPMO: lytic polysaccharide monooxygenase
LRR: lignin-rich residue from birch
SE: steam-explosion
VS: volatile-solids
