# Supplementary material for: The use of lytic polysaccharide monooxygenases in anaerobic digestion of lignocellulosic materials
Source: Biotechnol Biofuels. 2019 Nov 16;12:270. doi: 10.1186/s13068-019-1611-8 (PMC6858683; doi:10.1186/s13068-019-1611-8)
Supplement: Supplementary file 1 — Additional file 1: Figure S1. (A) Chromatogram of soluble fractions of anaerobic digestion reactions with Avicel supplemented with purified NcLPMO9C as well as H2O2. Samples were taken initially in reactions supplemented with purified NcLPMO9C, after addition of H2O2 or H2O. Shoulder peaks representing Glc4gemGlc, seen in reactions with NcLPMO9C only, are highlighted by the dashed lines. (B) Chromatogram of a standard sample (0.001 g / L) obtained after treating cellopentaose with NcLPMO9C, run together with other samples. Figure S2. Rate of methane accumulation during anaerobic digestion of Avicel with addition of enzymes Cellic Ctec2 (A), Celluclast (B), NcLPMO9C (C) and a blend of 85 % Celluclast and 15 % NcLPMO9C (D). Enzymes were supplied once at day 0 at 4 mg of protein per gram of substrate, with or without following addition of H2O2, as indicated in the figure. Hydrogen peroxide was supplied at 0h, 24h, 48h and 72h at 0.1 mM final concentration. Deionized water was added in all reactions without H2O2. Control reactions with boiled enzyme are also shown. Methane release by the inoculum methane was subtracted from all samples prior to calculation of methane production. The curves represent the average of two separate experiments. Figure S3. Rate of methane accumulation during anaerobic digestion of birch with addition of enzymes Cellic Ctec2 (A), Celluclast (B), NcLPMO9C (C) and a blend of 85 % Celluclast and 15 % NcLPMO9C (D). Enzymes were supplied once at day 0 at 4 mg of protein per gram of substrate, with or without following addition of H2O2, as indicated in the figure. Hydrogen peroxide was supplied at 0h, 24h, 48h and 72h at 0.1 mM final concentration. Deionized water was added in all reactions without H2O2. Control reactions with boiled enzyme are also shown. Methane release by the inoculum methane was subtracted from all samples prior to calculation of methane production. The curves represent the average of two separate experiments. Figure S4. Rate of [file 13068_2019_1611_MOESM1_ESM.docx]

**The use of lytic polysaccharide monooxygenases in anaerobic digestion of lignocellulosic materials**

Thales H.F. Costa^1^; Vincent G.H. Eijsink^1^; Svein Jarle Horn^1*^

1) Faculty of Chemistry, Biotechnology and Food Science, Norwegian University of Life Sciences (NMBU), P.O. Box 5003, N-1432 Aas, Norway

*Corresponding author

**Additional file**


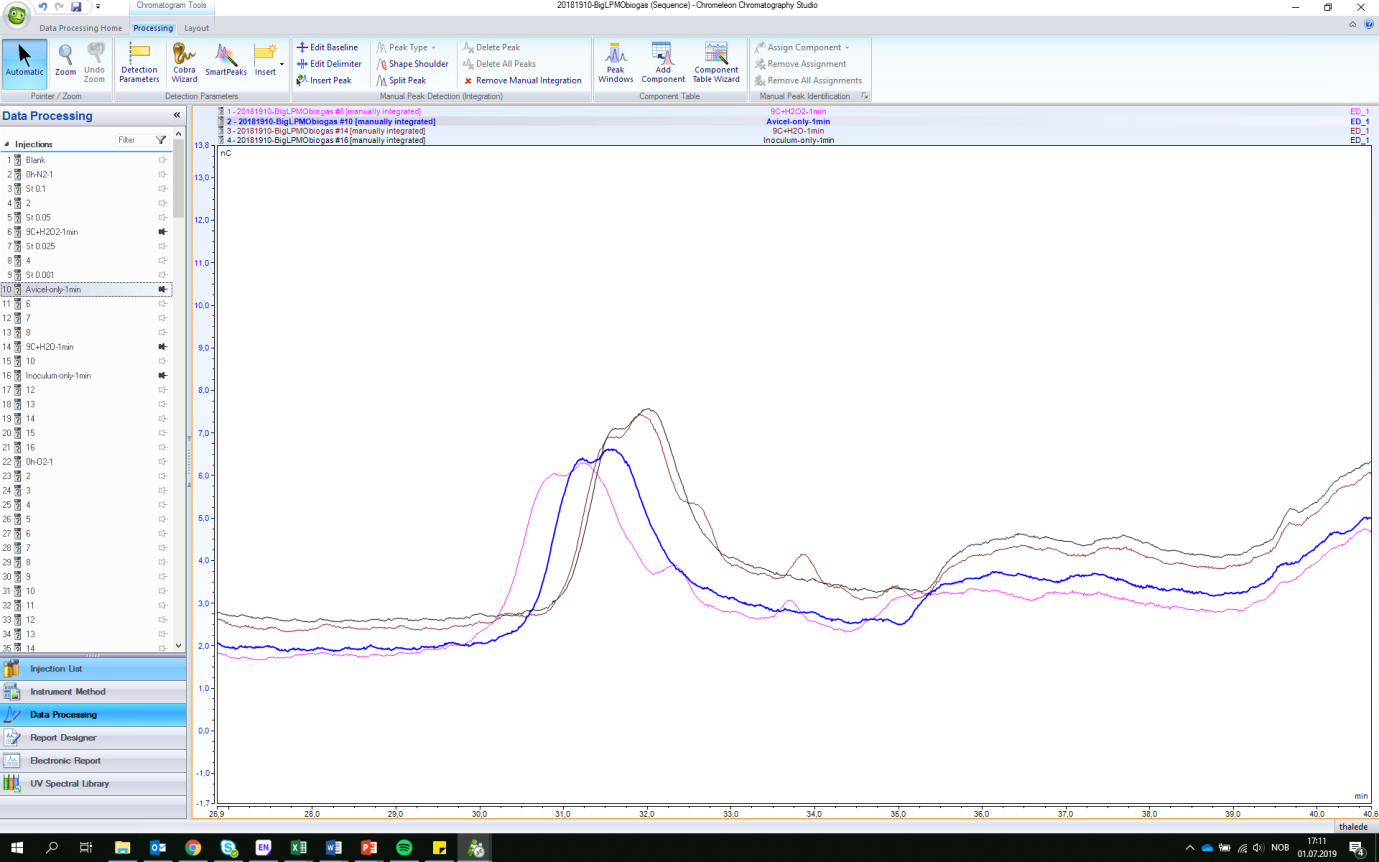


**A**

**Inoculum-only**

**Avicel-*Nc*LPMO9C**

**Avicel-only**

**Avicel-*Nc*LPMO9C+H2O2**


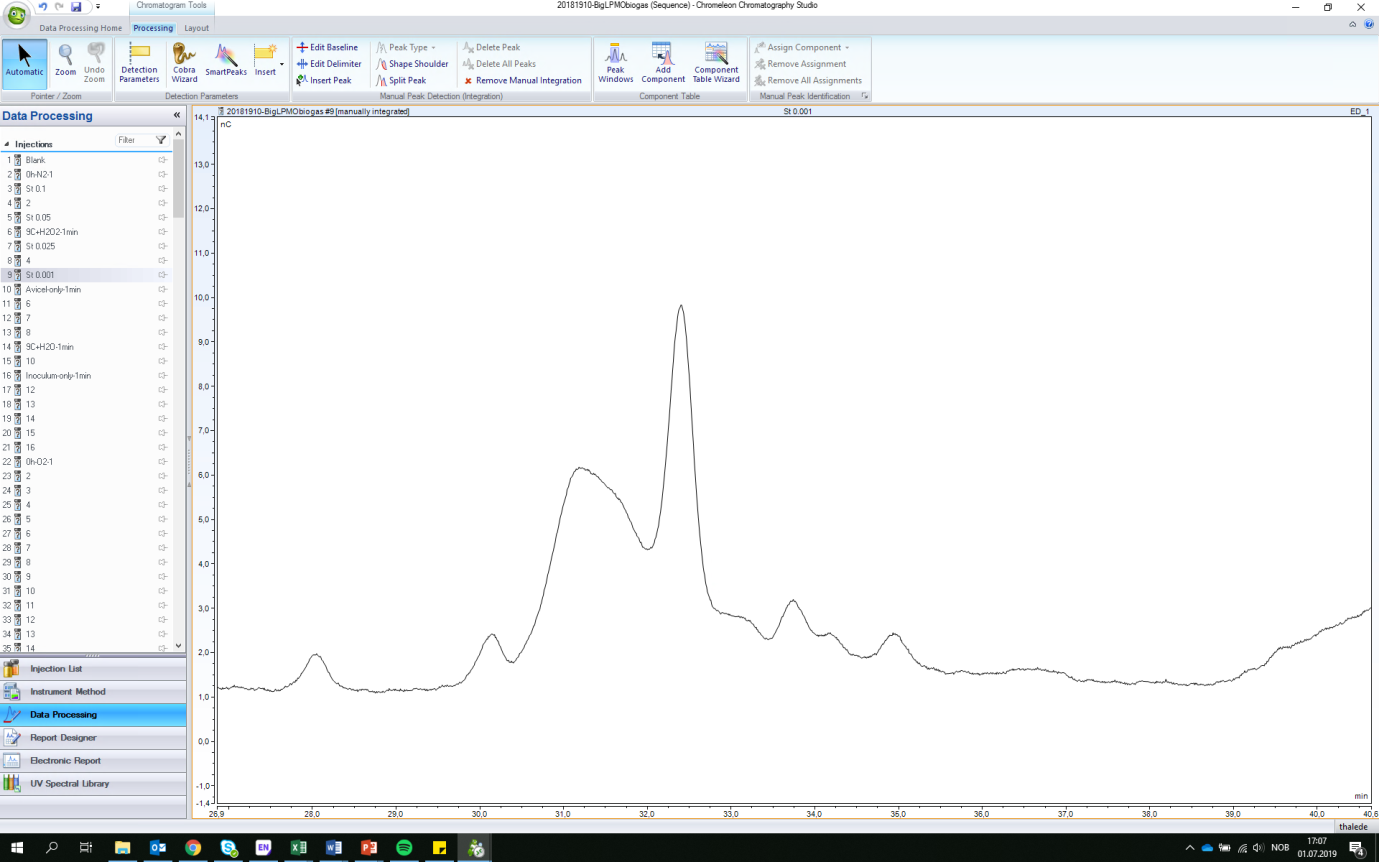


**B**

**Positive control**

**Glc4gemGlc – 0.001g/L**

**Figure S1.** (A) Chromatogram of soluble fractions of anaerobic digestion reactions with Avicel supplemented with purified *Nc*LPMO9C as well as H_2_O_2_. Samples were taken initially in reactions supplemented with purified *Nc*LPMO9C, after addition of H_2_O_2_ or H_2_O. Shoulder peaks representing Glc4gemGlc, seen in reactions with *Nc*LPMO9C only, are highlighted by the dashed lines. (B) Chromatogram of a standard sample (0,001 g/L) obtained after treating cellopentaose with *Nc*LPMO9C, run together with other samples.

**B**

**C**

**A**

**D**

**Figure S2.** Rate of methane accumulation during anaerobic digestion of Avicel with addition of enzymes Cellic Ctec2 (A), Celluclast (B), *Nc*LPMO9C (C) and a blend of 85 % Celluclast and 15 % *Nc*LPMO9C (D). Enzymes were supplied once at day 0 at 4 mg of protein / g of substrate, with or without following addition of H_2_O_2_, as indicated in the figure. Hydrogen peroxide was supplied at 0h, 24h, 48h and 72h at 0.1 mM final concentration. Deionized water was added in all reactions without H_2_O_2_. Control reactions with boiled enzyme are also shown. Methane release by the inoculum methane was subtracted from all samples prior to calculation of methane production. The curves represent the average of two separate experiments.

**B**

**A**

**C**

**D**

**Figure S3.**  Rate of methane accumulation during anaerobic digestion of birch with addition of enzymes Cellic Ctec2 (A), Celluclast (B), *Nc*LPMO9C (C) and a blend of 85 % Celluclast and 15 % *Nc*LPMO9C (D). Enzymes were supplied once at day 0 at 4 mg of protein / g of substrate, with or without following addition of H_2_O_2_, as indicated in the figure. Hydrogen peroxide was supplied at 0h, 24h, 48h and 72h at 0.1 mM final concentration. Deionized water was added in all reactions without H_2_O_2_. Control reactions with boiled enzyme are also shown. Methane release by the inoculum methane was subtracted from all samples prior to calculation of methane production. The curves represent the average of two separate experiments.

**B**

**A**

**C**

**D**

**Figure S4.**  Rate of methane accumulation during anaerobic digestion of spruce with addition of enzymes Cellic Ctec2 (A), Celluclast (B), *Nc*LPMO9C (C) and a blend of 85 % Celluclast and 15 % *Nc*LPMO9C (D). Enzymes were supplied once at day 0 at 4 mg of protein / g of substrate, with or without following addition of H_2_O_2_, as indicated in the figure. Hydrogen peroxide was supplied at 0h, 24h, 48h and 72h at 0.1 mM final concentration. Deionized water was added in all reactions without H_2_O_2_. Control reactions with boiled enzyme are also shown. Methane release by the inoculum methane was subtracted from all samples prior to calculation of methane production. The curves represent the average of two separate experiments.

**B**

**C**

**A**

**D**

**Figure S5.** Rate of methane accumulation during anaerobic digestion of lignin-rich residue from birch (LRR) with addition of enzymes Cellic Ctec2 (A), Celluclast (B), *Nc*LPMO9C (C) and a blend of 85 % Celluclast and 15 % *Nc*LPMO9C (D). Enzymes were supplied once at day 0 at 4 mg of protein / g of substrate, with or without following addition of H_2_O_2_, as indicated in the figure. Hydrogen peroxide was supplied at 0h, 24h, 48h and 72h at 0.1 mM final concentration. Deionized water was added in all reactions without H_2_O_2_. Control reactions with boiled enzyme are also shown. Methane release by the inoculum methane was subtracted from all samples prior to calculation of methane production. The curves represent the average of two separate experiments.

**Figure S6.**  Control reactions showing cumulative methane production during anaerobic digestion of Bovine Serum Albumin (BSA) with and without addition of H_2_O_2_ (methane production from inoculum-only has been substracted). As for all the other reactions with enzyme addition, the load of protein at day 0 were 9.2 mg/L of protein. H_2_O_2_ was supplied at 0h, 24h, 48h and 72h at 0.1 mM final concentration. Deionized water was added in all reactions without H_2_O_2_. The methane production was divided by the same amount of substrate (0.07 g VS) used in all reactions with lignocellulosic substrates for direct comparison. The curves represent the average of two separate experiments.


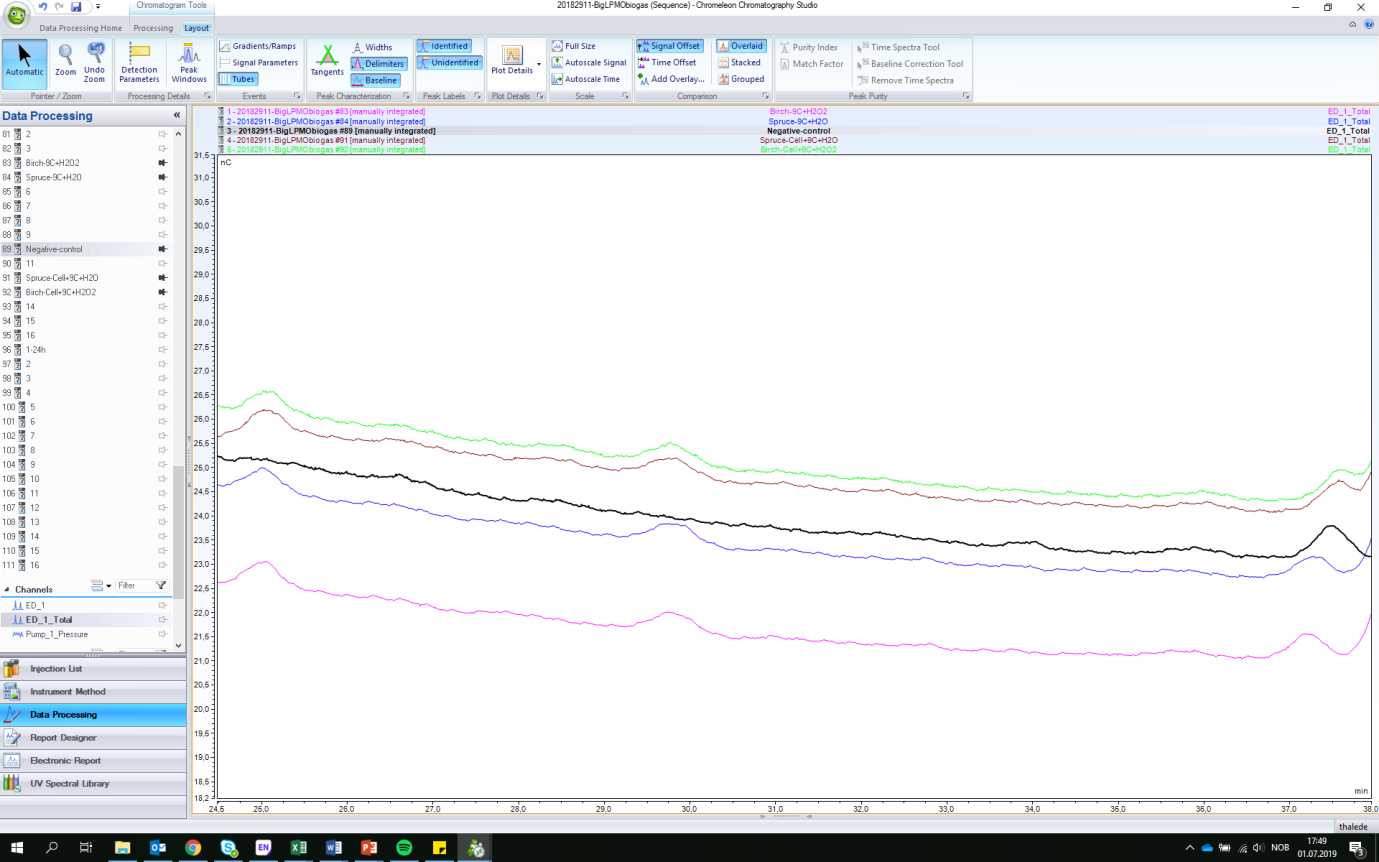


**A**

**Birch-Cell+9C+H_2_O_2_**

**Spruce-Cell+9C**

**Negative control**

**Spruce-*Nc*LPMO9C**

**Birch-*Nc*LPMO9C+H_2_O_2_**


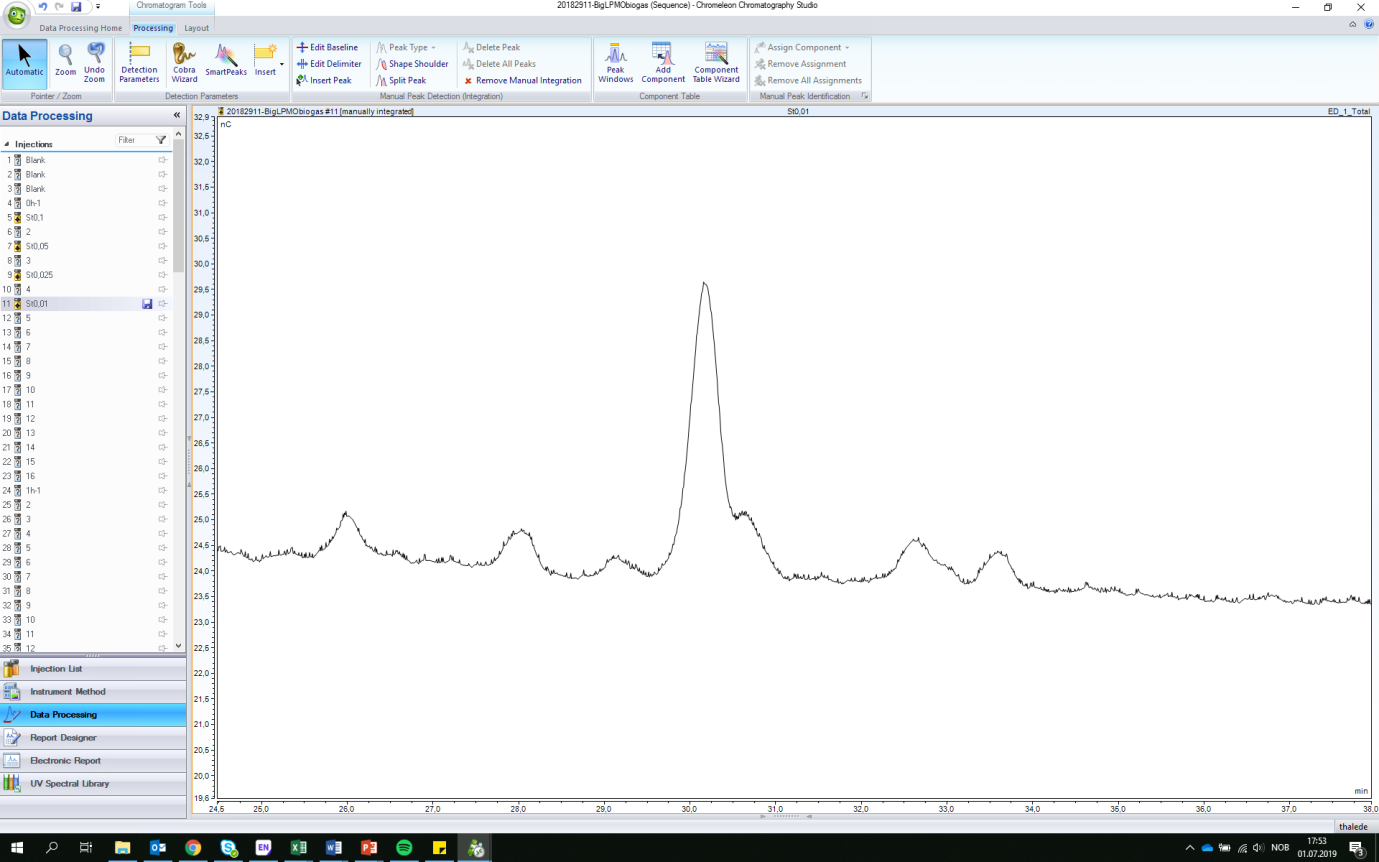


**B**

**Positive control**

**Glc4gemGlc – 0.001g/L**

**Figure S7.** (A) Chromatogram of soluble fractions of anaerobic digestion reactions with birch and spruce, supplemented with purified *Nc*LPMO9C or the blend of 85 % Celluclast and 15 % *Nc*LPMO9C (Cell+9C) as well as H_2_O_2_ or H_2_O, under anaerobic conditions. The samples shown were taken after 4h of incubation from reactions. Peaks representing Glc4gemGlc for this run are highlighted by the dashed lines. (B) Chromatogram of a standard sample (0,001 g/L) obtained after treating cellopentaose with *Nc*LPMO9C.
